# Low-Molecular-Weight PEGs for Cryopreservation of Stem Cell Spheroids

**DOI:** 10.34133/bmr.0037

**Published:** 2024-06-06

**Authors:** Madhumita Patel, Brent Vernon, Byeongmoon Jeong

**Affiliations:** ^1^ Department of Chemistry and Nanoscience, Ewha Womans University, 52 Ewhayeodae-gil, Seodaemun-gu, Seoul 03760, Republic of Korea.; ^2^ School of Biological and Health Systems Engineering, Arizona State University, Tempe, AZ 85287-9709, USA.

## Abstract

Stem cell spheroids (SCSs) are a valuable tool in stem cell research and regenerative medicine. SCSs provide a platform for stem cell behavior in a more biologically relevant context with enhanced cell–cell communications. In this study, we investigated the recovery of SCSs after cryopreservation at –196 °C for 7 days. Prior to cryopreservation, the SCSs were preincubated for 0 h (no preincubation), 2 h, 4 h, and 6 h at 37 °C in the presence of low-molecular-weight poly(ethylene glycol) (PEG) with molecular weights of 200, 400, and 600 Da. The recovery rate of SCSs was markedly affected by both the PEG molecular weight and the preincubation time. Specifically, when SCSs were preincubated with a PEG200 solution for 2 to 6 h, it significantly enhanced the recovery rate of the SCSs. Internalization of PEG200 through simple diffusion into the SCSs may be the cryoprotective mechanism. The PEG200 diffuses into the SCSs, which not only suppresses osmotic pressure development inside the cell but also inhibits ice formation. The recovered SCSs demonstrated both fusibility and capabilities for proliferation and differentiation comparable to SCSs recovered after dimethyl sulfoxide 10% cryopreservation. This study indicates that PEG200 serves as an effective cryoprotectant for SCSs. A simple preincubation procedure in the presence of the polymer greatly improves the recovery rate of SCSs from cryopreservation.

## Introduction

Stem cells offer important potential in regenerative medicine, biomedical research, and personalized healthcare. With their remarkable ability to differentiate into various cell types, they are invaluable for treating various diseases and injuries. Recently, stem cell spheroids (SCSs) have gained prominence due to their distinct advantages over stem cells cultured in 2-dimensional plates. These advantages include improved cell survival, enhanced proliferation, expression of stemness-related genes, and angiogenic and anti-inflammatory properties [1–3]. Spheroids are 3-dimensional cellular aggregates, offering a closer proximity to essential features found in living tissue, facilitating cell–cell interactions and cell–extracellular matrix interactions [4]. SCSs have various applications, from drug discovery and disease modeling to tissue engineering and regenerative medicine [5–9]. SCSs have also been used for cardiovascular and musculoskeletal tissue regeneration [10,11]. However, the practical application of stem cells and their spheroids often hinges on their long-term storage and accessibility. Cryopreservation, the process of freezing and storing cells at ultra-low temperatures, has proven to be an effective strategy for the long-term preservation of cells [12]. Dimethyl sulfoxide (DMSO) is the most commonly used cryoprotectant. Typically, a system with 10% DMSO in a media is used. DMSO protects cells by dehydrating them, replacing intracellular water, and reducing osmotic shock [13,14]. However, DMSO is cytotoxic and has detrimental effects such as membrane disruption, protein denaturation, global DNA methylation, and histone acetylation [12,15]. DMSO can also be involved in stem cell post-differentiation [16]. Therefore, new biocompatible cryoprotectants have been extensively investigated, mostly at the single-cell level [17–22]. Cryopreservation of cell spheroids might provide important information of the cryopreservation of higher level biosystems such as tissues and organs; however, it has rarely been reported [23–25]. Even when cryopreservation of separate cells works well by using cryoprotectants, significant challenges still remain for cryopreservation of cell spheroids due to their intricate 3-dimensional structures and the potential susceptibility to damage caused by ice crystal formation inside the core of the spheroids during freezing and thawing [23]. Overcoming these challenges requires careful selection of cryoprotectants and optimization of freezing/thawing methods to ensure the viability and functionality of the recovered cell spheroids. Bissoyi et al. [23] introduced polyampholytes with DMSO (10%) to improve the outcome of HepG2 cell spheroid cryopreservation. Similarly, in the presence of DMSO, pollen-washing water increased HepG2 and adenocarcinoma human alveolar basal epithelial cell (A549) spheroid recovery from cryopreservation by inhibiting intracellular ice formation [24]. A recent study by Matsumura et al. [25] also used polyampholytes to improve the recovery rate of SCSs. These cryoprotectants alone were not self-sufficient for preserving the spheroids and they used DMSO as a co-cryoprotectant; therefore, the search for a new biocompatible and cyto-compatible cryoprotectant for cell spheroids is still a crucial issue.

Polyethylene glycol (PEG) is a safe substance for human use and has been found to be effective in cryopreserving bacteriophages, microorganisms, and individual cells [26–29]. When added with DMSO during cryopreservation, PEG has also been found to improve the survival of mouse and porcine oocytes [30,31]. Recently, PEG coacervate was reported as a very effective cryoprotectant for stem cells [21]. Although the exact mechanism of how PEG works in freezing is not yet fully understood, it is known that PEGs with high molecular weight cannot enter the cell and lower the freezing point of solutions. The extracellular PEGs promote the dehydration of cells through osmotic pressure and thereby reduce intracellular ice formation, preventing cryoinjury [30,32]. PEG suppresses lipid peroxidation and stabilizes the membrane during subzero preservation [33–35]. Recent studies have shown that the effectiveness of PEGs in cryopreservation depends on their molecular weights [29,36]. The intracellular uptake of low-molecular-weight PEGs through diffusion contributes to the inhibition of ice recrystallization and exhibit excellent cryoprotecting properties for stem cells at the single-cell level [29].

In this study, we investigated whether low-molecular-weight PEGs can also be used as a cryoprotectant for SCSs. We first prepared SCSs and cryopreserved them in liquid nitrogen (–196 °C) for 7 days after preincubation using low-molecular-weight PEG (200, 400, and 600 Da).We preincubate the SCSs for 0 h (no preincubation), 2 h, 4 h, and 6 h at 37 °C in the presence of the PEGs, then examined them for recovery from the cryopreservation. In addition, the fusibility and capability for proliferation/differentiation of the SCSs were investigated to confirm the healthy state of the recovered SCSs.

## Materials and Methods

### Materials

PEGs (200 Da: TCI, Republic of Korea; 400 Da: TCI, Republic of Korea; 600 Da and PEG20K Da: Sigma Aldrich, USA) were used as received. In this paper, PEG200, PEG400, PEG600, and PEG20K are employed for PEGs with respective molecular weights of 200, 400, 600, and 20K Da. Cell culture media (Dulbecco’s modified Eagle medium [DMEM]) and supplements like fetal bovine serum (FBS), penicillin, and streptomycin were purchased from Corning, USA.

### Stem cell culture and spheroid formation

Ewha Womans University Medical School, based in Seoul, Republic of Korea, kindly donated the tonsil tissue-derived stem cells used in this study. The cells were obtained according to the NIH guidelines. These cells were cultured in DMEM supplemented with 10% (v/v) FBS, 1% (v/v) antibiotic–antimitotic, and 1% (v/v) penicillin/streptomycin in a sterile incubator at 37 °C under a 5% CO_2_ atmosphere. The cells were cultured up to passage 6 and used for spheroid formation. The confluent cells were collected using trypsin and then seeded at a density of 1 × 10^6^ cells per spheroid dish (SPL, Republic of Korea). The dishes were placed in an incubator at 37 °C for 24 h. The next day, the SCSs with a diameter of 80 to 150 μm were collected in phosphate-buffered saline (PBS) and then used for the cryopreservation study.

### Cryopreservation and cell recovery

The spheroids were equally divided (~300) into cryovials (Thermo Scientific Nunc, USA) containing 0.5 ml of a specific PEG solution chosen from PEG200, PEG400, or PEG600, all prepared at a concentration of 10 wt.% in DMEM. Subsequently, the vials were incubated at 37 °C under 5% CO_2_ conditions for 0 h (no incubation), 2 h, 4 h, and 6 h. The cryovials were transferred to a cryo box (Mr. Frosty; Thermo Scientific) and the vials were slowly cooled to −80 °C at 1 °C/min in the controlled freezing container. Following a 12-h incubation, the cryovials were transferred to liquid nitrogen (–196 °C) and stored for 7 days. A solution containing 10% DMSO in DMEM (DMSO 10%) was used as a positive control, whereas DMEM alone was used as a negative control for cryopreservation of the SCSs. The cryovials were removed from the liquid nitrogen and thawed using a 37 °C water bath. The spheroids were then moved to a conical tube and diluted 10 times with a DMEM growth medium. The SCSs were collected by centrifugation at 1,500 rpm for 5 min, and the supernatant was removed. The SCSs were then resuspended in 500 μl of growth media. Next, the viability of the spheroids was assessed using a live/dead assay kit (Invitrogen, USA) and a cell counting kit-8 (CCK-8; Dojindo, Japan).

### Cytotoxicity of low-molecular-weight PEG

The cytotoxicity of PEG200, PEG400, and PEG600 at 10 wt.% in DMEM was evaluated for SCSs in suspension state under culture conditions. The SCSs suspended in solution were incubated for 0 h, 2 h, 4 h, and 6 h at 37 °C and then analyzed using a live/dead assay and CCK-8 kits.

### Ice recrystallization inhibition

Aqueous PEG solutions (10 wt.% in DMEM) were dropped from 1.4 m onto a slide glass on aluminum foil cooled to dry ice (–78 °C). The frozen droplets formed a thin ice wafer, which was annealed at –6 °C for 30 min in a cryostage. The splat assay is widely used for ice recrystallization inhibition (IRI) assay [17,18,20,23]. The images were taken through crossed polarizers using a microscope (CX40IT, Soptop, China) equipped with a 10×/0.25/∞/-/FN26.5 lens (UIS-2, Olympus Ltd., Japan) and a 3M CMOS color digital camera (BoliOptics, USA). The average crystal size was calculated by using ImageJ. DMEM without PEGs was used as a control and assigned to be 100% in the mean largest grain size (MLGS) analysis.

### Ice nucleation inhibition

Aqueous solutions of PEG (10 wt.%) were filtered through a poly(vinylidene fluoride) filter with a pore size of 0.22 μm, manufactured by Whatman, Fisher Scientific, USA. Then, 20 droplets (0.5 μl/droplet) of each PEG solution were pipetted onto the cryostage (LTS120, Linkam Scientific Instruments Ltd, UK). The cryostage was cooled to 5 °C at a rate of –30 °C/min and kept at this temperature for 3 min. Then, the sample was cooled to –30 °C with a cooling rate of –1 °C/min. The frozen fraction of the droplets was monitored, and the nucleation temperatures at which half of the droplets were frozen were recorded [29].

### PEG internalization

SCSs were suspended in a DMEM solution containing PEG to investigate the internalization mechanism. The SCSs were treated separately with inhibitors of macropinocytosis (5.0 μg/ml rottlerin), caveolae-mediated endocytosis (5.0 μg/ml filipin), and clathrin-mediated endocytosis (15.0 μg/ml chlorpromazine) for 30 min [29,37,38]. Afterward, the cells were treated with PEG200 and PEG20K at 100 μM for an additional 6 h at 37 °C. SCSs were then collected by centrifugation at 1,500 rpm for 5 min and washed with PBS 3 times. Next, spheroids were resuspended in 400 μl of PBS and ultrasonicated using an Ultrasonic Processor from Sonics and Materials Inc. USA for 1 min at 30 amp. Subsequently, the samples were centrifuged at 9,000*g* for 20 min to remove cellular debris and the supernatant was collected. The intracellular PEGs collected from the supernatant were quantified using the enzyme-linked immunosorbent assay (ELISA) kit for PEG from MyBioSource, USA.

### SCS diffusion model

To compare the expected uptake of PEGs assuming passive diffusion for both, the time-dependent concentration of PEGs in the SCS was modeled using non-dimensionalized Fick’s second law in spherical coordinates (Eq. 1) in Matlab using relative diffusion coefficients 0.16 (for PEG20K), 0.65 (for PEG400), 0.76 (for PEG400), and 1 (for PEG200). The Matlab code is provided in the Supplementary Materials. The simulated loading in each SCS as a function of time was found by integrating the concentration of the PEG over the volume of the SCS at each time using Eq. 2.

$$\frac{dC^{*}}{dt^{*}}=\frac{D^{*}}{r^{*2}}\left(\frac{\partial }{\partial r^{*}}\left(r^{2}\frac{\partial C^{*}}{\partial r^{*}}\right)\right)$$ (1)

$$M=4\pi \int_{0}^{\mathrm{Ro}}C^{*}\left(r^{*}\right)\;r^{*2}dr^{*}\;$$ (2)

C^*^ = *C*(*t*)/*C*_o_, where *C*(*t*) is the concentration at each position *r* at each time and *C_o_* is the loading concentration of the PEG; D* = *D*/*D*_o_ is the relative diffusion coefficient where *D* is the diffusion coefficient for the PEG into the SCS and *D*_o_ is the diffusion coefficient for PEG200 into the SCS; r* = *r*/*R*_o_, where *r* is the radial position in the SCS and *R*_o_ is the radius of the SCS. t* = *t*/*t*_o_ is the non-dimensional time where *t* is the actual diffusion time and *t*_o_ = *R*_o_^2^/*D*. Finally, relative time was found for each PEG using *t*^#^ = t*D* to reconstitute the difference in diffusion coefficients in the non-dimensional time.

### Evaluation of cytoskeleton

Cytoskeletal integrity of SCSs was studied using phalloidin staining agents (Abcam, USA). The recovered SCSs were fixed with 4% formaldehyde for 15 min and then washed twice with PBS. After fixing, SCSs were permeabilized with 0.25% Triton X-100 in PBS for 10 min and stained with phalloidin-iFluor 488 reagents (1/1,000) for 30 min. Then, SCSs were stained with 4′,6-diamidino-2-phenylindole, and fluorescence images were taken using a Zeiss LSM 880 confocal microscope (Carl Zeiss, Germany).

### Spheroid proliferation and fusibility of SCSs

The recovered SCSs were seeded in a 96-well plate for proliferation studies. The cell images were obtained using a live/dead assay kit after 3 days of proliferation. The cells were treated with a solution of ethidium homodimer-1 (2.0 μM) and calcein AM (2.0 μM) for 30 min. The fluorescence images were captured using an Olympus IX71 fluorescence microscope and Olympus DP2-BSW software. For quantitation of cell proliferation using CCK-8 analysis, the growth medium was replaced by a CCK-8 solution (10% in DMEM containing 1% [v/v] penicillin/streptomycin) and incubated for 150 min at 37 °C. The absorbance was measured at 450 nm relative to 655 nm to quantitatively assess the cell proliferation. For this assay, 100% was assigned to the data obtained at 0 days (3 h).

Additionally, SCS fusibility was studied. The recovered SCSs were seeded in 96-U-ultra low attachment plates (Thermo Fisher Scientific, USA), and cultured with 100 μl of growth medium for 3 days, and images of SCSs fusion were observed.

### Assessment of stem cell differentiation

The potential of differentiation into osteo-, chondro-, and adipogenic lineages of the recovered SCSs was studied. The SCSs were seeded in a 24-well plate, and the media were replaced every 2 days. Once the cells reached confluence, they were treated with specific osteo-, chondro-, and adipogenic induction media for 2 weeks while the media were replaced every 2 days [3]. Then, the cells were fixed with 4% formaldehyde, washed with PBS, and stained with alizarin red, alcian blue, and oil red O for osteo-, chondro-, and adipogenic differentiation, respectively. The images were captured using an Olympus IX71 fluorescence microscope and Olympus DP2-BSW software.

### Statistical analysis

Experiments were conducted in triplicate for statistical accuracy. The significance of the data was determined via one-way ANOVA with Tukey tests. Any differences with a *P* value less than 0.05 and 0.01 were considered statistically significant and marked as * and **, respectively.

## Results

### SCS preparation

**Fig. 1.**
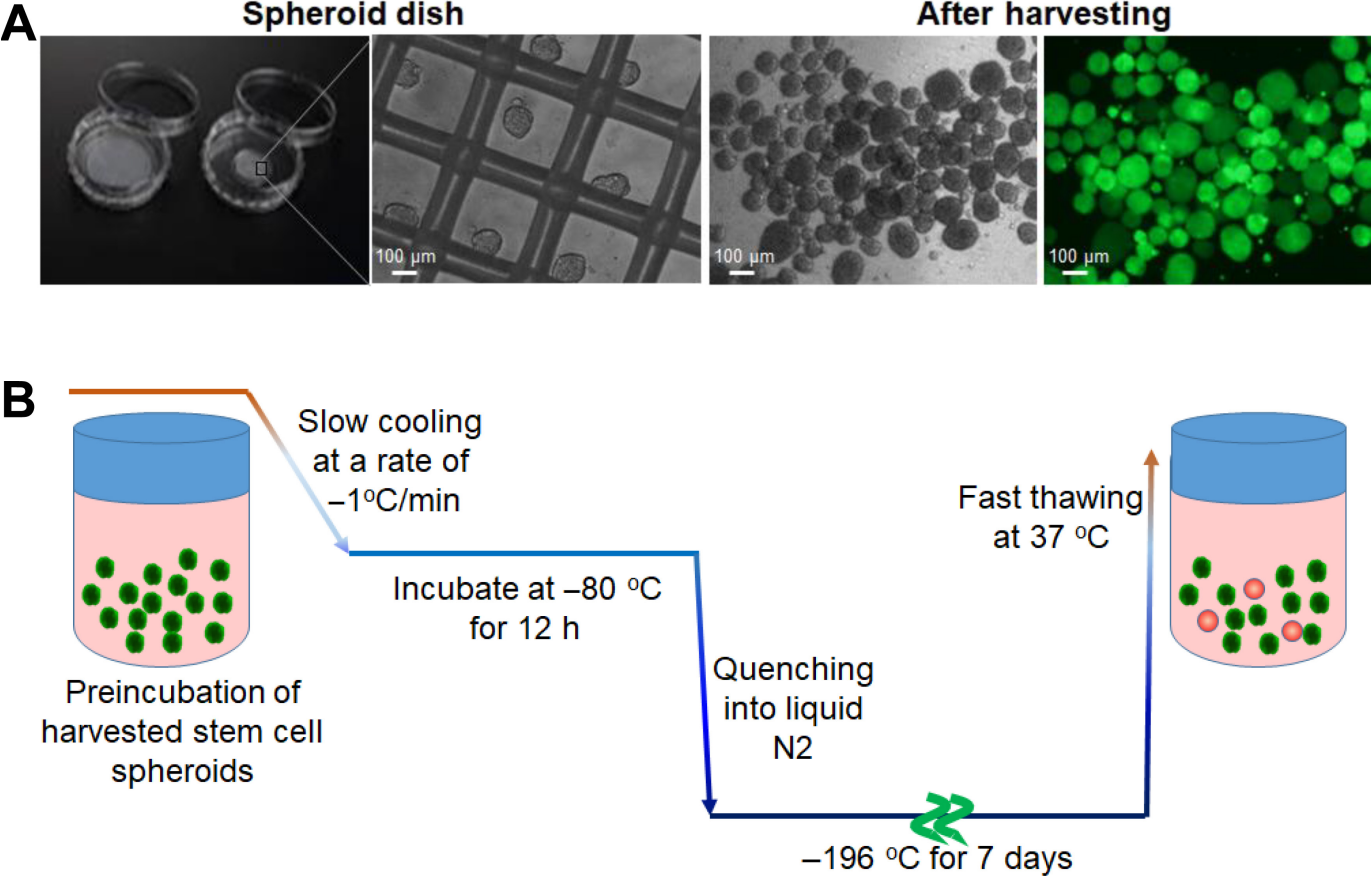
(A) Preparation of SCSs. Phase-contrasted images and live/dead images of SCSs are shown in white/black and color, respectively. The green fluorescence indicates live cells. (B) Cryopreservation process of harvested SCSs. The SCSs were suspended in DMEM solutions containing low-molecular-weight PEGs and preincubated for 0 to 6 h before cryopreservation. They were slowly cooled to –80 °C at a cooling rate of –1 °C/min and incubated at –80 °C for 12 h. Then, they were quenched into liquid nitrogen and cryopreserved at –196 °C for 7 days, followed by fast thawing at 37 °C.

SCSs with 80 to 150 μm size were prepared using a cell spheroid dish. The spheroid size varies depending on the number of cells cultured on the spheroid dish. With an optimized number of 1 × 10^6^ cells per dish, spheroids of size 80 to 150 μm are formed. The live/dead images of harvested SCSs indicate that all SCSs were healthy as shown in green (Fig. 1A). After the preincubation of the SCSs for 0 to 6 h in DMEM solutions containing PEGs with molecular weights of 200, 400, and 600 Da, the SCSs were cryopreserved at –196 °C for 7 days according to a slow-freezing protocol (Fig. 1B) [16,38].

### SCS recovery from cryopreservation

**Fig. 2.**
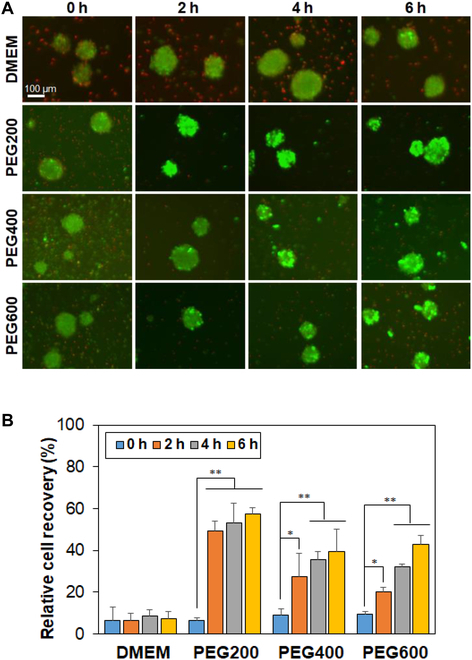
(A) Live/dead images of SCSs recovered from cryopreservation at –196 °C for 7 days. Live and dead cells are shown in green and red, respectively. 0 h, 2 h, 4 h, and 6 h indicate the preincubation time of SCSs in the presence of PEGs (10 wt.% in DMEM) at 37 °C before cryopreservation. The scale bar is 100 μm. (B) Quantitative analysis of spheroid recovery using the CCK-8 kit. Recovery rate of SCSs from cryopreservation in DMSO 10% was assigned as 100%. *N* = 3. The asterisks * and ** indicate *P* < 0.05 and *P* < 0.01, respectively.

Recovery of SCSs after cryopreservation was significantly affected by preincubation time and molecular weight of the PEG. The live/dead images of the recovered SCSs taken immediately after thawing are shown in Fig. 2A. The images using a traditional cryopreservation medium (DMSO 10% in DMEM) are shown in Fig. S1. The population of dead cells stained in red significantly decreased by preincubation of the SCSs in low-molecular-weight PEG solutions before cryopreservation. In addition, many dead cells were observed following cryopreservation when the SCSs were preincubated in the DMEM without PEG, irrespective of incubation time. In contrast, the live cells stained in green exhibited significantly high fluorescence intensity for SCSs recovered from cryopreservation after preincubation with PEG200 for 2 to 6 h. SCSs preincubated in PEG400 and PEG600 for 4 to 6 h also showed increased fluorescence intensity indicating live cells (green); however, the results were not as good as that obtained with PEG200. The quantitative analysis of SCS recovery from cryopreservation was performed by the CCK-8 kit (Fig. 2B). For DMEM, the recovery rate was low irrespective of incubation time over 6 h, while PEG200 improved the recovery rate from 6% to 49%, 53%, and 57% with the incubation time of 0 (no incubation), 2 h, 4 h, and 6 h, respectively. On the other hand, the recovery rate increased to 27% → 35% → 39% for the PEG400 system and to 20% → 32% → 42% for the PEG600 system by preincubation for 2 h, 4 h, and 6 h, respectively. For PEG200, there is no statistical difference among 2 to 6 h, suggesting that 2 h preincubation is enough to provide the cell recovery. In the current study, PEG200 exhibited the highest cell recovery and thus we focused on the PEG200. As for the PEG400 and PEG600, more than 6 h might be required to improve the cell recovery.

### Cytotoxicity of low-molecular-weight PEGs

**Fig. 3.**
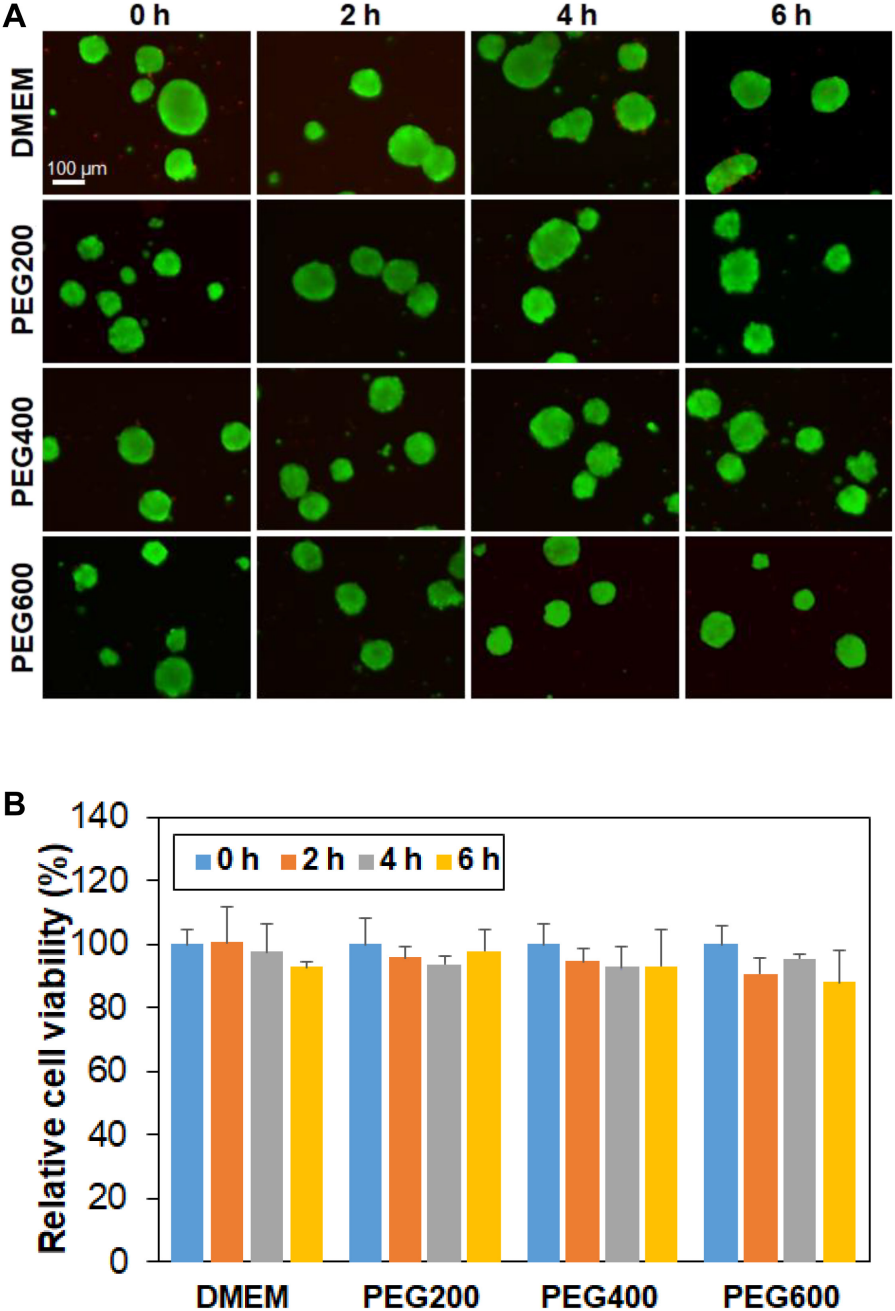
Cytotoxicity assay of PEGs for SCSs. (A) Live/dead images of SCSs in DMEM containing PEGs. The SCSs were suspended in the solution and incubated at 37 °C for 0 h, 2 h, 4 h, and 6 h. The scale bar is 100 μm. (B) Cell viability assayed using the CCK-8 kit. The relative cell viability was compared with 0 h (100%).

To understand how the preincubation period and PEG molecular weight affect the behavior of cell recovery, we checked several possibilities. First, the cytotoxicity of PEGs was evaluated during preincubation. After the preincubation over 0 to 6 h, the live/dead images of SCSs are shown in Fig. 3A. The preincubated SCSs remained intact as stained in green. This fact indicates that the PEG solutions (10 wt.%) are not toxic for the SCSs within the preincubation time over 0 to 6 h. The quantitative results measured with the CCK-8 assay kit also suggested that the SCSs survived well during the preincubation period over 0 to 6 h in PEG solutions (10 wt.% in DMEM) at 37 °C before cryopreservation (Fig. 3B).

### IRI and INI of low-molecular-weight PEGs

**Fig. 4.**
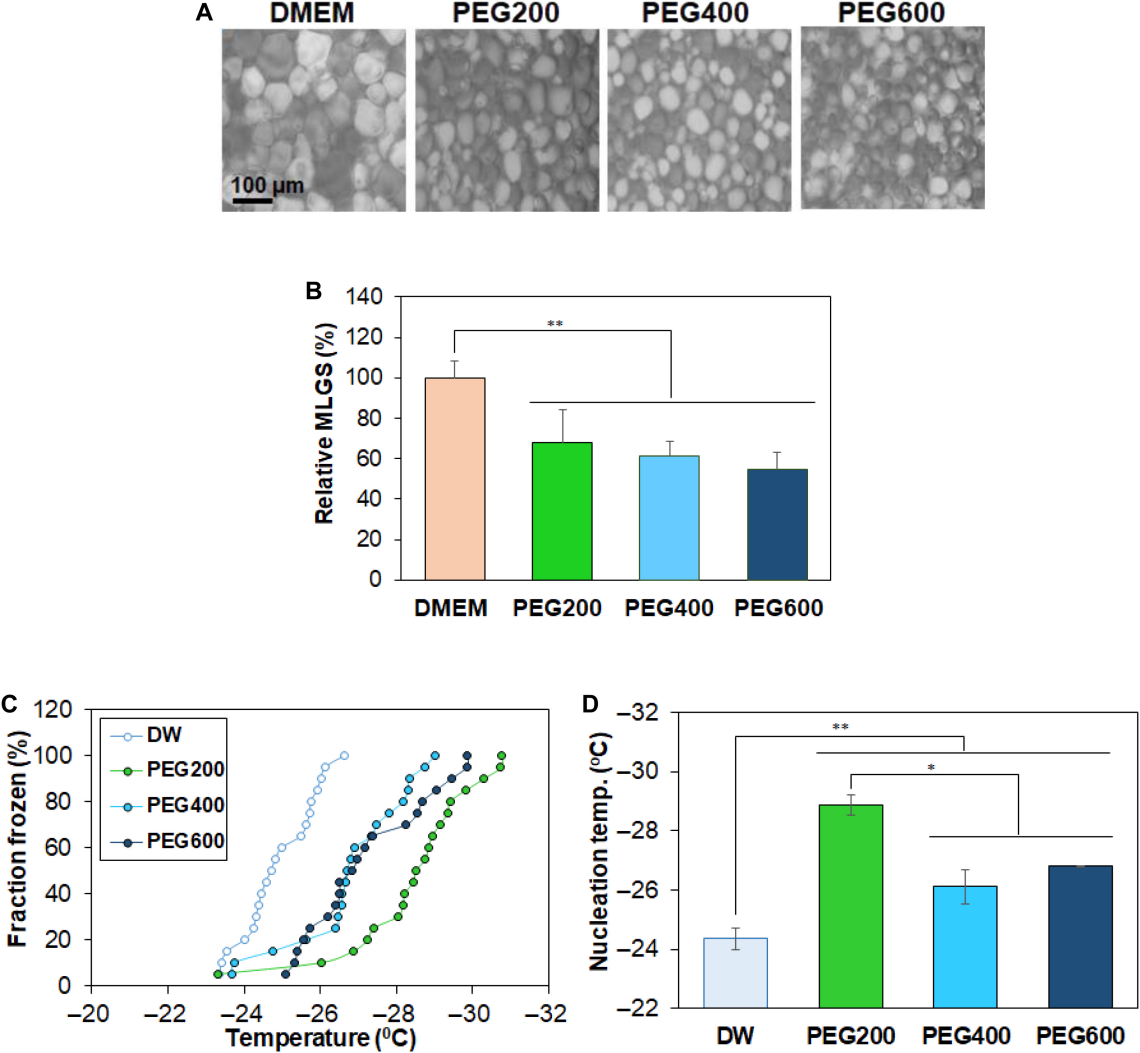
(A) Ice crystal images formed from PEG aqueous solution (10.0 wt.% in DMEM). The images of ice crystals formed from DMEM without PEG were also shown as a control. (B) Mean largest grain size (MLGS) of PEG aqueous solutions (10 wt.% in DMEM). The MLGS of ices formed from DMEM without PEG was assigned to be 100%. (C) INI measurements of PEG aqueous solutions (10 wt.%). Twenty droplets were used for determination of INI activity. (D) Ice nucleation temperature at which 50% of the droplets were frozen.

The IRI and ice nucleation inhibition (INI) were measured as they are well-known protection mechanisms of cryoprotecting agents [39]. Ice crystal images formed from aqueous PEG solutions (10 wt.% in DMEM) indicated that the ice crystal size decreased in the presence of PEGs (Fig. 4A). Semiquantitative analysis of the ice crystal size was performed by measuring the MLGS. It indicated that the ice crystal size decreased to 68%, 61%, and 55% of that of DMEM in the presence of PEG200, PEG400, and PEG600, respectively (Fig. 4B). PEGs also inhibited ice nucleation. The frozen fraction significantly decreased in the PEG solutions, compared with water (Fig. 4C). However, PEG200 is more effective at depressing water’s freezing point than PEG400 and PEG600 at the same concentration. The nucleation temperature defined as the temperature at which half of the droplets was frozen decreased from –24 °C (deionized water, DW) to –28 °C for PEG200 and –26 °C for both PEG400 and PEG600 (Fig. 4D). The greatest decrease of the ice nucleation temperature was observed for PEG200.

### Internalization of PEGs into the SCSs

**Fig. 5.**
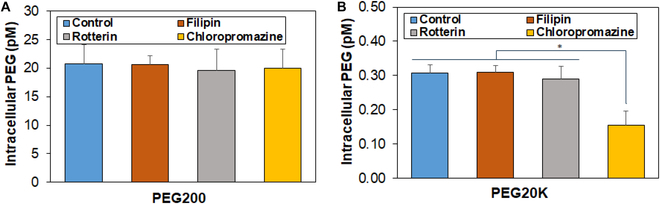
Internalization of PEG200 (A) and PEG20K (B) into the SCSs. SCSs were incubated for 6 h in the PEG solutions (10 wt.%) in DMEM in the presence of inhibitors. The control indicates the same protocol in the absence of inhibitors. *N* = 3. No statistical significance in the internalization of PEG200 among the treated systems in (A). The asterisk * in (B) indicates *P* < 0.05.

We quantified the intracellular PEG content using a PEG ELISA kit (Fig. 5). Specifically, we compared PEG200 with PEG20K. Permeation of PEGs through the plasma membrane depends on the PEG molecular weight. PEGs with a molecular weight less than 2K Da enter the cell by passive diffusion, while those with a molecular weight greater than 5K Da enter the cell via a combination of passive diffusion and caveolae-mediated endocytosis [36]. SCSs were treated without inhibitors (control) and inhibitors of rottlerin, filipin, and chlorpromazine, which are inhibitors for macropinocytosis, caveolae-mediated endocytosis, and clathrin-mediated endocytosis, respectively [29]. Then, SCSs were treated with PEGs. No difference was observed between PEG200 with and without inhibitor treatments, suggesting that PEG200 enters the cells via passive diffusion (Fig. 5A). On the other hand, a significant decrease in intracellular PEG20K were observed with the treatment of chlorpromazine, indicating that clathrin-mediated endocytosis is a main mechanism of internalization into the cells for the PEG20K (Fig. 5B).

### SCS diffusion model

**Fig. 6.**
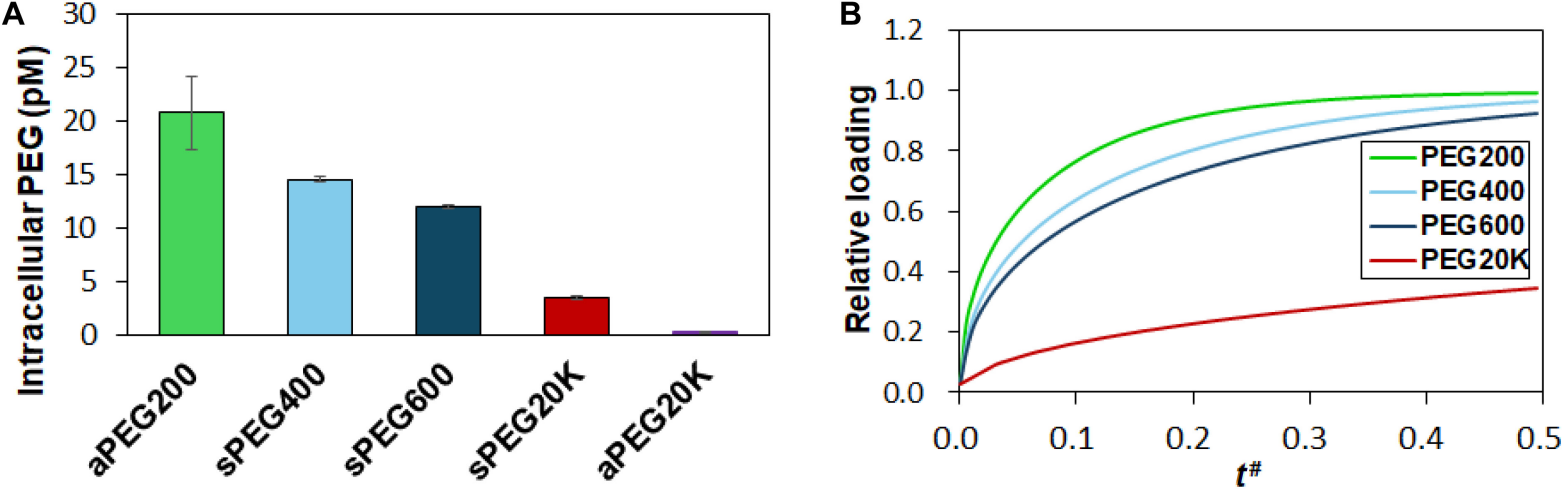
(A) The simulated internalization for PEGs compared to PEG200 assuming passive diffusion. The simulated data were obtained using Fick’s law. The observed actual PEG200 and PEG20K internalization is shown (aPEG200 and aPEG20K) in comparison. aPEG and sPEG indicate actual and simulated PEG, respectively. (B) Simulated relative loading of PEGs in SCS of equivalent diameters versus relative time (*t*^#^) assuming non-dimensional diffusion coefficients with a ratio of 1/0.76/0.65/0.16 for PEG200/PEG400/PEG600/PEG20K.

The diffusion coefficient (*D*) of a permeant is known to be a function of molecular weight as described by Eq. 3, and the hydrodynamic radius (*R*_H_) of PEG in water can be described by the regression model shown in Eq. 4 [40,41].

$$D=\mathrm{kT}/\left(6\pi \eta R_{H}\right)$$ (3)

$$R_{H}=0.06127M^{0.3931}$$ (4)

where *k*, *T*, and *η* are the Boltzmann constant, temperature, and viscosity of medium in the system. *M* is the molecular weight of the PEG. Based on the above Eq. 4, the *R*_H_ of PEG200, PEG400, PEG600, and PEG20K are calculated to be 0.49, 0.65, 0.76, and 3.01 nm, respectively. Therefore, the ratios of diffusion coefficients of PEGs, that is, *D*_PEG200_/*D*_PEG400_/*D*_PEG600_/*D*_PEG20K_ from Eq. 1 is 1/0.76/0.65/0.16 at a constant temperature. The expected intracellular uptake of PEGs compared to the actual PEG200 uptake was modeled using Fick’s law assuming that the primary mechanism in both cases was passive diffusion (Fig. 6A). Figure 6B presents the time courses of simulated internalization for PEG400, PEG600, and PEG20K compared to the actual PEG200 uptake assuming passive diffusion for all. For reference, the figure also shows the actual PEG20K internalization. This deviation from the model for the actual PEG20K supports the argument that at this molecular weight, another mechanism is driving the internalization other than passive diffusion. The difference in diffusion of PEGs among PEG200, PEG400, and PEG600 must be involved in the difference in cell recovery rate of SCSs from cryopreservation.

### F-actin polymerization of recovered SCSs from cryopreservation

**Fig. 7.**
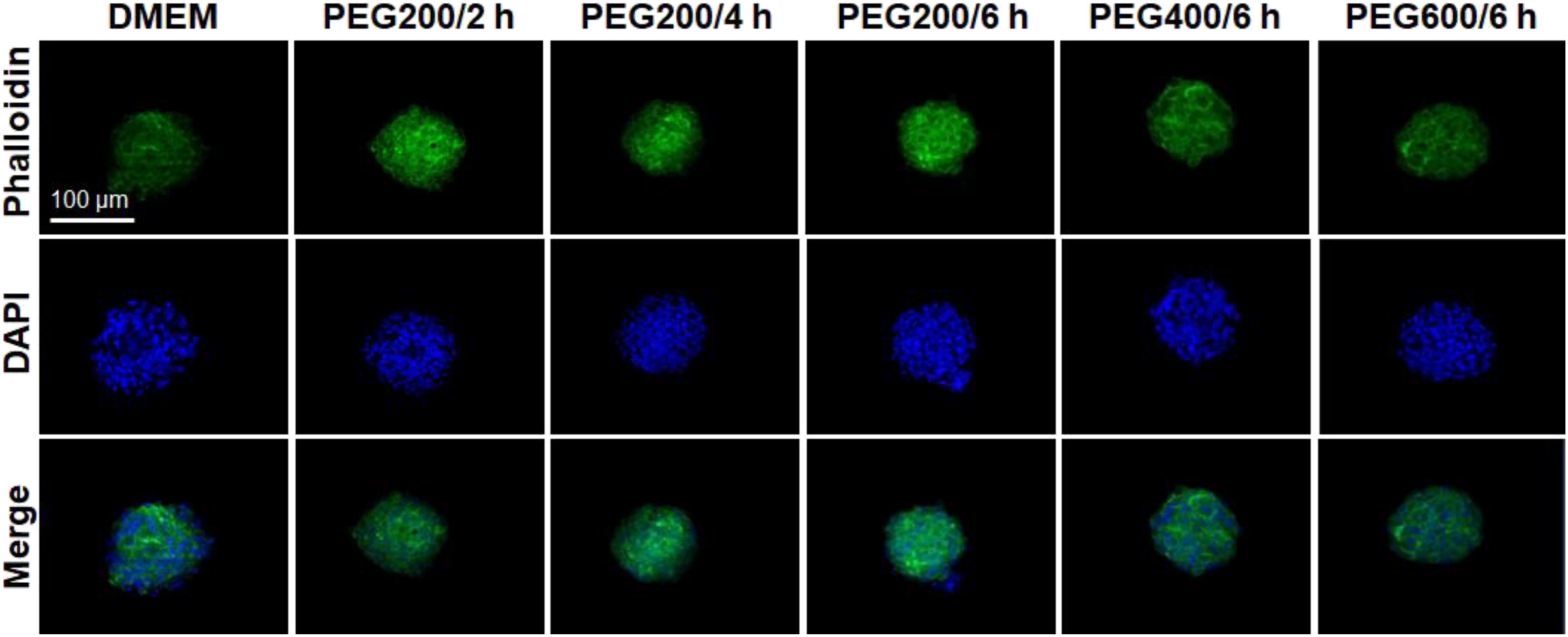
F-actin (green) staining of SCSs recovered from cryopreservation. The scale bar is 100 μm. The PEG200/6 h indicates the SCSs preincubated at 37 °C for 6 h before cryopreservation.

The healthy state of the recovered SCSs from cryopreservation was confirmed by F-actin polymerization, fusibility, proliferation, and differentiation of the SCSs. F-actin is critical in cell adhesion, migration, proliferation, differentiation, and maintaining cell integrity [42,43]. Recovered SCSs were stained with phalloidin to label polymerized F-actin filaments and the cytoskeletal integrity was investigated. The fluorescence intensity of green color indicates the level of F-actin. The cells cryopreserved after the preincubation using PEG200 over 2 to 6 h exhibited that F-actin level was higher than control (DMEM) without using PEGs (Fig. 7), but similar to that of using DMSO 10% (Fig. S2). SCSs preincubated using PEG400 and PEG600 for 6 h exhibited the lower levels of F-actin staining than those preincubated using PEG200 for 6 h, indicating that PEG200 saved the post-thaw actin polymerization function of SCSs from cryoinjury more effectively than PEG400 and PEG600 [23].

### Fusibility of recovered SCSs from cryopreservation

**Fig. 8.**
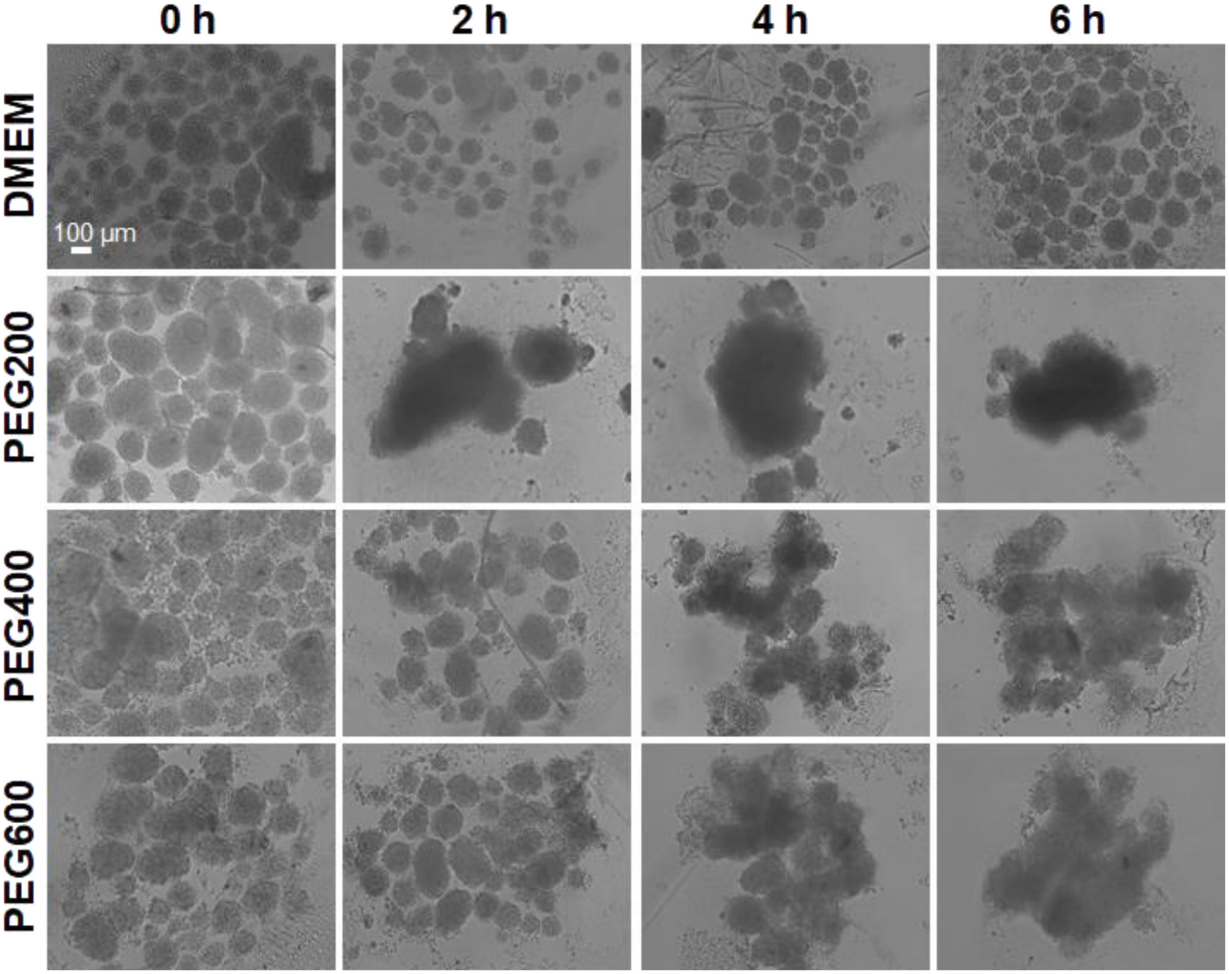
Fusibility of the recovered SCSs using PEG200, PEG400, and PEG600 preincubation for 0 to 6 h followed by cryopreservation at –196 °C for 7 days. SCSs were put together on a non-adhesive dish for fusibility, and their fusion was observed in 3 days. The scale bar is 100 μm.

Organ regeneration is based on a biological and biophysical process of tissue fusion. Hence, we assessed one of the roles of the SCSs, i.e., their ability to fuse together as a measure of healthy state of the SCSs [44]. Recovered SCSs from cryopreservation were assayed for their fusibility for 3 days post-thaw. SCSs with a higher bioactivity fused more quickly [45]. The SCSs preincubated with PEGs exhibited good fusibility (Fig. 8). The control SCSs recovered from cryopreservation after preincubation using DMEM without PEGs did not exhibit any fusibility, similar to 0-h incubation systems. The PEG200-preincubated SCSs exhibited the highest fusibility among all the systems investigated in this study. The levels of fusibility of SCSs preincubated with PEG200 were as good as that of SCSs recovered from cryopreservation using the traditional method (DMSO 10%) (Fig. S3).

### Proliferation and differentiation of recovered SCSs from cryopreservation

**Fig. 9.**
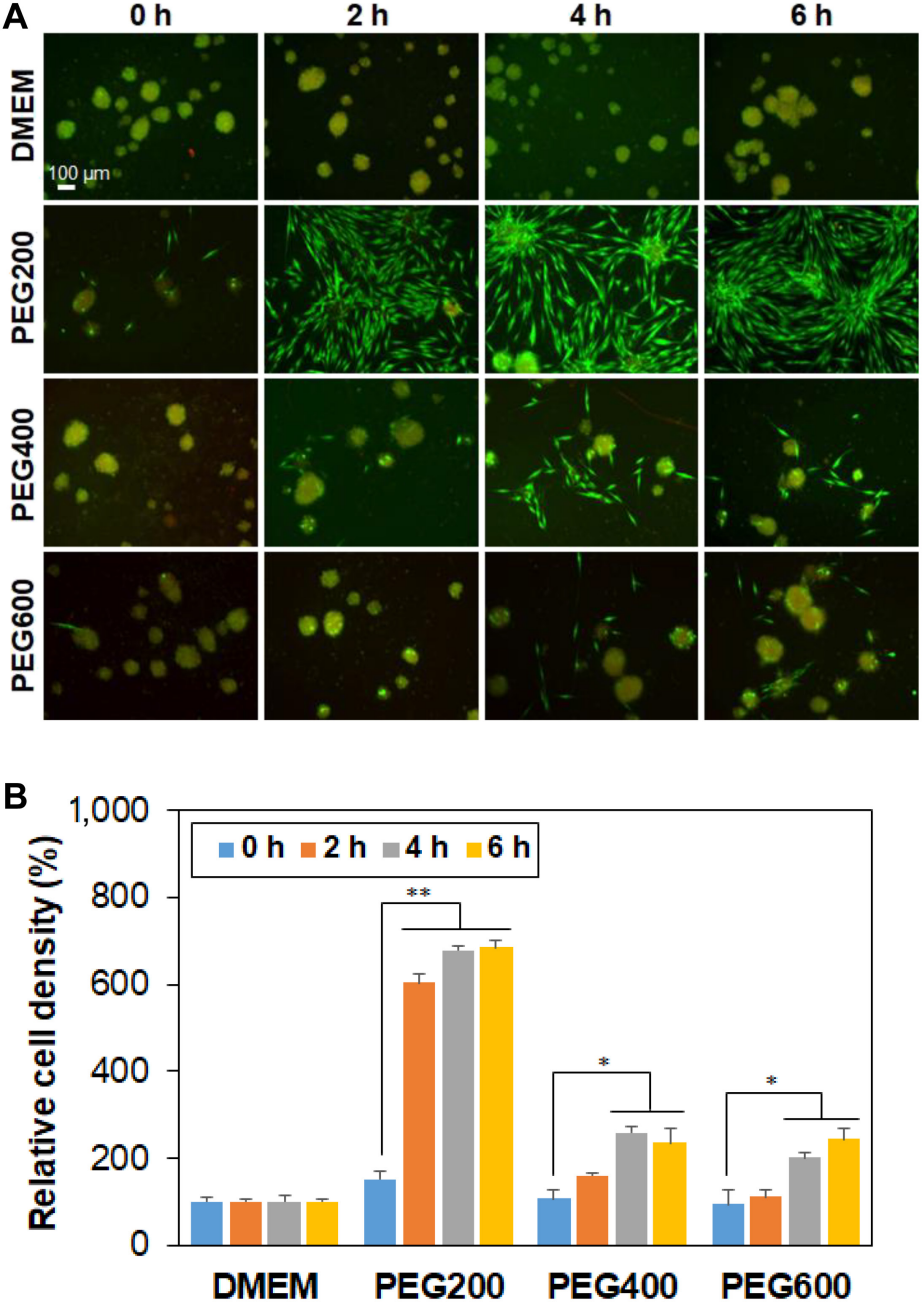
Proliferation of SCSs recovered from cryopreservation at –196 °C for 7 days. (A) Fluorescence images of cells 3 days after the proliferation. The scale bar is 100 μm. (B) Quantitative analysis of cell proliferation relative to day 0 (100%) assayed by the CCK-8. *N* = 3. The asterisks * and ** indicate *P* < 0.05 and *P* < 0.01, respectively.

SCSs recovered from cryopreservation were studied for proliferation using 96-well culture plates (traditional adhesive culture plates) to confirm their healthy state. Only the SCSs preincubated using PEG200 over 2 to 6 h and those using the traditional method (DMSO 10%) disintegrated from the spheroids and proliferated well in the plates (Fig. 9A and Fig. S4). On the other hand, SCSs preincubated with PEG400 and PEG600 showed very poor proliferation. SCSs without PEG preincubation (0 h) exhibited little or no proliferation or disintegration of SCSs. Neither proliferation nor cell adhesion was observed for the SCSs cryopreserved using DMEM only. The quantitative assay also confirmed the good proliferation of the PEG200-preincubated system (Fig. 9B). The cell proliferation study suggested that PEG200 preincubation over 2 to 6 h is the only recommendable protocol to lead healthy cells.

**Fig. 10.**
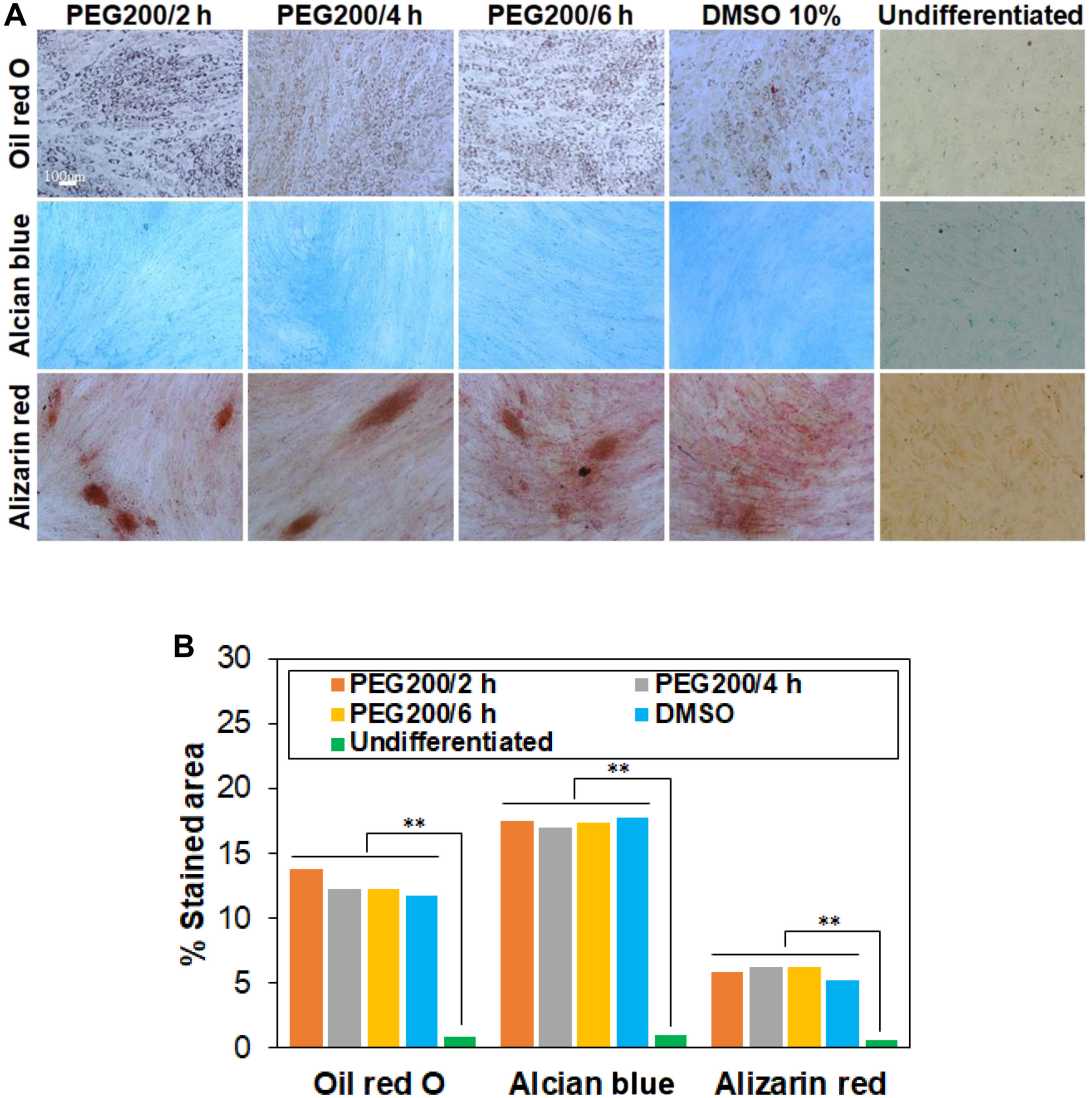
Differentiation of recovered SCSs into osteocytes, chondrocytes, and adipocytes. (A) The cells were stained in red, blue, and brown by oil red O (adipogenic), alcian blue (chondrogenic), and alizarin red (osteogenic), respectively. (B) Semiquantitative expression of the images using ImageJ software.

The capability toward adipogenic, chondrogenic, and osteogenic differentiation of recovered SCSs from cryopreservation was evaluated by oil red O, alcian blue, and alizarin red staining, respectively. Lipids/triglycerides stained in red (oil red O), sulfated proteoglycans stained in blue (alcian blue), and calcium ions stained in brown (alizarin red) are measures of the differentiation capability of the stem cells [3,46]. We selected PEG200-preincubated SCSs over 2 to 6 h and proliferated them in an adhesive plate to reach confluency because only the system exhibited high cell proliferation rate. Then, the stem cells were driven to undergo differentiation in each induction medium. The stem cells undergo adipogenic, chondrogenic, and osteogenic differentiation, indicating the healthy state of the SCSs (Fig. 10A). The images were analyzed using ImageJ software and the percentage of the stained area was semi-quantitatively expressed (Fig. 10B).

All these results suggest that preincubation of PEG200 has the potential for higher cell recovery, proliferation, fusion, and differentiation ability.

## Discussion

PEGs have been reported as a cryoprotectant of bacteria, mammalian cells, mouse oocytes, and porcine oocytes at the single-cell level [28–31]. In addition, our recent study also proved that single-cell recovery from cryopreservation was significantly increased by preincubation of stem cells with low-molecular-weight PEGs [29]. Cryopreservation of SCSs is a more challenging issue than that of single cells due to the large size and complex 3-dimensional structures of the SCSs, which demands transport processes of cryoprotectants and control of ice formation throughout the SCSs. Here, we hypothesized that the preincubation of SCSs in the presence of low-molecular-weight PEGs at 37 °C just before the cryopreservation might improve recovery. SCSs with 80 to 150 μm size were prepared because the size of SCSs affects the PEG internalization as well as cell recovery.

Cell recovery from the cryopreservation strongly suggest that preincubation of SCSs in the presence of low-molecular-weight PEGs, in particular PEG200, significantly improved the recovery rate of SCSs from the cryopreservation. Aqueous solutions (10 wt.%) of all low-molecular-weight PEGs including PEG200, PEG400, and PEG600 were not toxic for the SCSs within the preincubation time over 0 to 6 h. Our previous research also suggested that diffusion is a primary mechanism of internalization of PEG600 into stem cells at the single-cell level [29]. These results and our current inhibitor studies using SCSs suggest that PEG200, PEG400, and PEG600 are internalized into the stem cells or SCSs by simple diffusion. However, a diffusion model study suggested the difference in diffusion of low-molecular-weight PEGs among PEG200, PEG400, and PEG600, which could affect the cell recovery rate of SCSs from cryopreservation.

In addition, the effectiveness of INI of PEG200 could contribute to the cell recovery of SCSs from the cryopreservation. Ice nucleation inhibition is related to the supercooling behavior of aqueous polymer solutions and is affected by interfacial tension, shape factor, and kinetic prefactors [47]. The ice nucleation temperature of an aqueous polymer solution usually decreases as the molecular weight of polymer increases, whereas this trend was true for PEGs when PEG molecular weight is greater than 600 [29,48]. The INI effect of aqueous PEG solutions measured using a differential scanning calorimeter (DCS) indicated that the ice nucleation temperature of aqueous PEG solutions (10 wt.%) lowered by 6.67, 3.40, 5.55, and 2.62 °C for PEG200, PEG300 and PEG400, and PEG600, respectively, compared with that of water without polymers. They suggested that colligative property effects worked and ice nucleation temperature decrease was greater for PEG200 than for PEG400 and PEG600. In addition, the Mark–Howink–Sakurada equation, [*η*] = *KM^a^* (*K* = 4.33 × 10^−4^ dl/g, *a* = 0.67) is obeyed when PEG molecular weight is greater than 600 [49]. The parameters correlate to the molecular size and shape based on the random coil conformation of polymer chains. In a low-molecular-weight region, the interactions between water molecules and PEG hydroxy end groups induce the deviation from the general trend of molecular weight effect on the physicochemical properties of PEGs, which are based on the random coil conformation of the polymers. Therefore, the INI effect of polymers can be discussed as follows. First, the nature of polymer affects the INI. PEG exhibited greater INI effect than poly(N-vinyl pyrrolidone) or dextran [48]. For example, aqueous solutions (10 wt.%) of PEG10K, PVP10K, and dextran10K exhibited ice nucleation temperature decrease of 5.48, 2.40, and 1.88 °C, respectively. Second, in relation to the nature of polymer, the hydroxy end groups of PEG become more significant as the molecular weight of PEG decreases. In addition, the ratio of the anti/gauche conformation of PEG can also be involved in INI of aqueous solutions of low-molecular-weight PEG, which is averaged for high-molecular-weight PEGs, and the trend for physicochemical properties based on random coil conformation of PEG can be consistently predicted above a certain molecular weight of PEGs. Third, freezing point decrease related to colligative property can be involved when discussing the effect of molecular weight on INI. At 10 wt.% aqueous polymer solution, for example, the molar concentration of PEG200 is 3 times higher than that of PEG600. Therefore, greater INI can be expected for lower-molecular-weight PEGs at the same weight-based concentration.

The post-thaw actin polymerization function and fusibility of SCSs indicates that the PEG200 preincubation over 2 to 6 h helps maintain the bioactivity of the recovered SCSs from the cryopreservation. Cell proliferation and differentiation studies also indicated the healthy state of the recovered SCSs from cryopreservation through preincubation of SCSs in the presence of PEG200.

SCSs were preincubated in PEG200, PEG400, and PEG600 solution (10 wt.%) over 0 to 6 h, and then cryopreserved at –196 °C for 7 days. PEG200 improved the recovery rate from 6% to 57% as the incubation time increased from 0 (no incubation) to 6 h. The recovery rate increased 39% and 42% for PEG400 system and the PEG600 system, respectively, when preincubated for 6 h. Live/dead images and CCK-8 assay proved that all PEGs are cyto-compatible for SCSs during the 6-h preincubation period in PEG solutions (10 wt.% in DMEM) at 37 °C. However, PEG200 is more effective in inhibiting ice nucleation than PEG400 and PEG600 at the same concentration. Inhibitor studies suggest that PEG200 enters the SCSs by passive diffusion. F-actin polymerization, fusibility, proliferation, and differentiation of recovered SCSs from cryopreservation indicated that PEG200 preincubation of SCSs over 2 to 6 h before cryopreservation lead to healthy states of the post-thaw stem cells more effectively than PEG400 and PEG600.

The presence of PEG in the SCSs may play a role in suppressing osmotic pressure and preventing ice recrystallization or nucleation. Our results indicate that preincubation of PEG200 over 2 to 6 h before cryopreservation is the effective protocol in SCS cryopreservation.

## Data Availability

Data are available on request from the authors.

## Supplementary Materials

Supplementary 1 Python code for diffusion model, live/dead images, and F-actin images of SCSs recovered from cryopreservation at –196 °C for 7 days using DMSO 10% in DMEM, fusibility of the SCSs at day 3, recovered from cryopreservation using DMSO 10% at –196 °C for 7 days, live/dead images of proliferated cells at day 3 of SCSs recovered from cryopreservation using DMSO 10%. Figs. S1 to S4
