# Supplementary material for: Low-Molecular-Weight PEGs for Cryopreservation of Stem Cell Spheroids
Source: Biomater Res. 2024 Jun 6;28:0037. doi: 10.34133/bmr.0037 (PMC11156479; doi:10.34133/bmr.0037)

**Supporting Information**

**Low Molecular Weight PEGs for Cryopreservation of Stem Cell Spheroids**

Madhumita Patel^1^, Brent Vernon^2*^, and Byeongmoon Jeong^1*^

1 Department of Chemistry and Nanoscience, Ewha Womans University, 52 Ewhayeodae-gil, Seodaemun-gu, Seoul 03760, Korea
2 School of Biological and Health Systems Engineering, Arizona State University, Tempe, AZ 85287-9709, USA

*Address correspondence to: [brent.Vernon@asu.edu](mailto:brent.Vernon@asu.edu), bjeong@ewha.ac.kr

**MATLAB CODE**

%% Fick's Second Law in spherical coordinates

clc; close all; clear all;

m = 2;

D = [1;0.76;0.65;0.16];

xend = 1;

tend = 0.5;

xres = 50;

tres = 100;

x = linspace(0,xend,xres);

t = linspace(0,tend,tres);

soln = pdepe(m,@pde,@ic,@bc,x,t);

Conc1 = soln(:,:,1);

Conc2 = soln(:,:,2);

Conc3 = soln(:,:,3);

Conc4 = soln(:,:,4);

%rmass1 = conctomass_over_time(Conc1,tres)

for t_index1 = 1:tres

mass_PEG200(t_index1) = trapz(x,4*pi*x.^2.*Conc1(t_index1,:));

loading_PEG200(t_index1) = mass_PEG200(t_index1)/((4/3)*pi*xend^3);

time_PEG200 = t./D(1);

end

for t_index2 = 1:tres

mass_PEG400(t_index2) = trapz(x,4*pi*x.^2.*Conc2(t_index2,:));

loading_PEG400(t_index2) = mass_PEG400(t_index2)/((4/3)*pi*xend^3);

load_ratio400(t_index2) = loading_PEG400(t_index2)/loading_PEG200(t_index2);

time_PEG400 = t./D(2);

end

for t_index3 = 1:tres

mass_PEG600(t_index3) = trapz(x,4*pi*x.^2.*Conc3(t_index3,:));

loading_PEG600(t_index3) = mass_PEG600(t_index3)/((4/3)*pi*xend^3);

load_ratio600(t_index3) = loading_PEG600(t_index3)/loading_PEG200(t_index3);

time_PEG600 = t./D(3);

end

for t_index4 = 1:tres

mass_PEG20K(t_index4) = trapz(x,4*pi*x.^2.*Conc4(t_index4,:));

loading_PEG20K(t_index4) = mass_PEG20K(t_index4)/((4/3)*pi*xend^3);

load_ratio20K(t_index4) = loading_PEG20K(t_index4)/loading_PEG200(t_index4);

time_PEG20K = t./D(4);

end

figure1 = figure;

plot(x,Conc1(2,:))

figure2 = figure;

plot(x,Conc2)

figure3 = figure;

plot(t,loading_PEG200)

hold on

plot(t,loading_PEG400)

hold on

plot(t,loading_PEG600)

plot(t,loading_PEG20K)

hold off

figure4 = figure;

plot(t,load_ratio400)

hold on

plot(t,load_ratio600)

plot(t,load_ratio20K)

hold off

[min_value_400, min_index_400] = min(load_ratio400);

[min_value_600, min_index_600] = min(load_ratio600);

[min_value_20K, min_index_20K] = min(load_ratio20K);

figure5 = figure;

t_common = linspace(0,0.5,100);

loading_PEG200_interp = interp1(time_PEG200, loading_PEG200,t_common,'linear','extrap');

loading_PEG400_interp = interp1(time_PEG400, loading_PEG400,t_common,'linear','extrap');

loading_PEG600_interp = interp1(time_PEG600, loading_PEG600,t_common,'linear','extrap');

loading_PEG20K_interp = interp1(time_PEG20K, loading_PEG20K,t_common,'linear','extrap');

plot(t_common,loading_PEG200_interp)

hold on

plot(t_common,loading_PEG400_interp)

plot(t_common,loading_PEG600_interp)

plot(t_common,loading_PEG20K_interp)

for i = 1:length(t_common)

load_ratio_int400(i) = loading_PEG400_interp(i)/loading_PEG200_interp(i);

load_ratio_int600(i) = loading_PEG600_interp(i)/loading_PEG200_interp(i);

load_ratio_int20K(i) = loading_PEG20K_interp(i)/loading_PEG200_interp(i);

end

hold off

figure6 = figure

hold on

plot(t_common,load_ratio_int400)

plot(t_common,load_ratio_int600)

plot(t_common,load_ratio_int20K)

hold off

[min_value_int400, min_index_int400] = min(load_ratio_int400)

[min_value_int600, min_index_int600] = min(load_ratio_int600)

[min_value_int20K, min_index_int20K] = min(load_ratio_int20K)

function [c,f,s] = pde(x,t,Conc,dudx)

D = [1;0.76; 0.65;0.16];

c = [1/D(1);1/D(2);1/D(3);1/D(4)];

f = [1;1;1;1].*dudx;

s = [0;0;0;0];

end

function u = ic(x)

Co = 0;

u = [Co;Co;Co;Co];

end

function [pl, ql, pr, qr] = bc(xl,ul,xr,ur,t,D,Co)

pl = [0;0;0;0];

ql = [1;1;1;1];

pr = [ur(1)-1;ur(2)-1;ur(3)-1;ur(4)-1];

qr = [0;0;0;0];

end

**Figure S1**. Live/dead images of stem cell spheroids (SCSs) recovered from cryopreservation at –196 ^o^C for 7 days using DMSO 10% in DMEM. The scale bar is 100 μm.


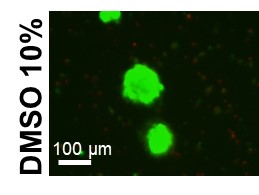


**Figure S2**. F-actin (green) images of stem cell spheroids (SCSs) recovered from cryopreservation at –196 ^o^C for 7 days using DMSO 10% in DMEM. The scale bar is 100 μm.


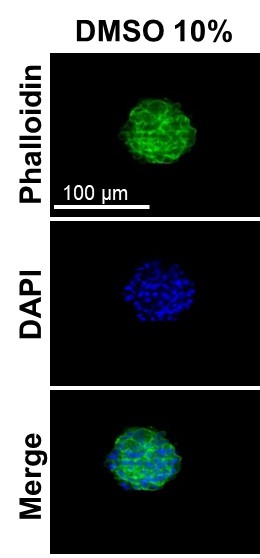


**Figure S3**. Fusibility of the stem cell spheroids at day 3, recovered from cryopreservation using DMSO 10% at –196 ^o^C for 7 days. The scale bar is 100 µm.


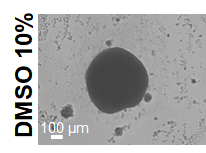


**Figure S4**. Live/dead images of proliferated cells at day 3 of SCSs recovered from cryopreservation using DMSO 10%. The scale bar is 100 µm.


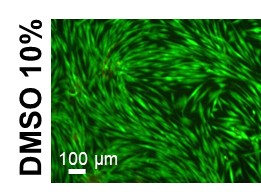

Supplement: Supplementary 1 — Python code for diffusion model, live/dead images, and F-actin images of SCSs recovered from cryopreservation at –196 °C for 7 days using DMSO 10% in DMEM, fusibility of the SCSs at day 3, recovered from cryopreservation using DMSO 10% at –196 °C for 7 days, live/dead images of proliferated cells at day 3 of SCSs recovered from cryopreservation using DMSO 10%. Figs. S1 to S4 [file bmr.0037.f1.docx]
